# The impact of multimodal analgesia based enhanced recovery protocol on quality of recovery after laparoscopic gynecological surgery: a randomized controlled trial

**DOI:** 10.1186/s12871-021-01399-2

**Published:** 2021-06-28

**Authors:** Zhiyu Geng, Hui Bi, Dai Zhang, Changji Xiao, Han Song, Ye Feng, Xinni Cao, Xueying Li

**Affiliations:** 1 Department of Anesthesiology, Peking University First Hospital, Beijing, China; 2 Department of Obstetrics and Gynecology, Peking University First Hospital, Beijing, China; 3 Department of Biostatics, Peking University First Hospital, Beijing, China

**Keywords:** Multimodal analgesia, Laparoscopy, Gynecological, Quality of recovery

## Abstract

**Background:**

Our objective was to evaluate the impact of multimodal analgesia based enhanced recovery protocol on quality of recovery after laparoscopic gynecological surgery.

**Methods:**

One hundred forty female patients scheduled for laparoscopic gynecological surgery were enrolled in this prospective, randomized controlled trial. Participants were randomized to receive either multimodal analgesia (Study group) or conventional opioid-based analgesia (Control group). The multimodal analgesic protocol consists of pre-operative acetaminophen and gabapentin, intra-operative flurbiprofen and ropivacaine, and post-operative acetaminophen and celecoxib. Both groups received an on-demand mode patient-controlled analgesia pump containing morphine for rescue analgesia. The primary outcome was Quality of Recovery-40 score at postoperative day (POD) 2. Secondary outcomes included numeric pain scores (NRS), opioid consumption, clinical recovery, C-reactive protein, and adverse events.

**Results:**

One hundred thirty-eight patients completed the study. The global QoR-40 scores at POD 2 were not significantly different between groups, although scores in the pain dimension were higher in Study group (32.1 ± 3.0 vs. 31.0 ± 3.2, *P* = 0.033). In the Study group, NRS pain scores, morphine consumption, and rescue analgesics in PACU (5.8% vs. 27.5%; *P* = 0.0006) were lower, time to ambulation [5.0 (3.3–7.0) h vs. 6.5 (5.0–14.8) h; *P* = 0.003] and time to bowel function recovery [14.5 (9.5–19.5) h vs.17 (13–23.5) h; *P* = 0.008] were shorter, C-reactive protein values at POD 2 was lower [4(3–6) ng/ml vs. 5 (3–10.5) ng/ml; *P* = 0.022] and patient satisfaction was higher (9.8 ± 0.5 vs. 8.8 ± 1.2, *P* = 0.000).

**Conclusion:**

For minimally invasive laparoscopic gynecological surgery, multimodal analgesia based enhanced recovery protocol offered better pain relief, lower opioid use, earlier ambulation, faster bowel function recovery and higher patient satisfaction, while no improvement in QoR-40 score was found.

**Trial registration:**

ChiCTR1900026194; Date registered: Sep 26,2019.

## Background

Enhanced recovery after surgery (ERAS) is a standardized, multidisciplinary protocol delivered to surgical patients aimed to reduce stress response, improve patient recovery and optimize surgical outcome after surgery. The ultimate endpoint of this program is to allow patients to restore functional capacity and resume daily activities rapidly. The ERAS protocols have been adopted for colorectal, gastric, urologic and pancreatic surgeries and improved patient outcomes including fewer complications, shorter length of hospital day and lower costs have been demonstrated [1–4].

Laparoscopic surgery is a minimally invasive procedure and an increasing number of gynecological surgeries are performed laparoscopically. Though less trauma injury, significant abdominal and shoulder pain is common during the early postoperative period and strong analgesics including opioids are required [5]. At the meantime, female patients undergoing laparoscopic surgery are high risk population for postoperative nausea and vomiting (PONV) and the incidence could be as high as 80% [6]. Multimodal pain management with nonopioids agents or regional anesthesia may improve analgesic efficacy and reduce opioids related adverse effects such as PONV. Hence, more benefit could be expected from multimodal pain management in this population.

Some retrospective studies have described the implementation of ERAS program for gynecological surgery and revealed outcomes focus on length of stay, narcotic requirement, and hospital cost. No randomized controlled trials have investigated the effect of an enhanced recovery pathway on patient quality of recovery (QoR) for laparoscopic gynecological surgery [7–10].

Thus, the primary goal of this randomized clinical trial was to investigate if multimodal analgesic regimen, as a part of enhanced recovery protocol, could improve patient recovery after gynecological laparoscopy. The Quality of Recovery 40 (QoR-40) questionnaire is a valid and reliable measurement used to assess the degree of recovery after different surgical types and anesthetic techniques. It provides a patient-assessed health status through five dimensions. We hypothesized that multimodal analgesic regimen would improve patient-reported recovery when compared with conventional pain control method. The primary outcome was QoR-40 score assessed at postoperative day (POD) 2. Secondary endpoints were morphine consumption, pain scores, time to functional recovery, serum marker of the surgical inflammatory response, and incidence of PONV during the first 48 h after surgery.

## Methods

This was a prospective, randomized clinical trial. This study was approved by the Ethics Review Board of Peking University First Hospital, Peking, China (No. 2019–173) in August 2019 and written informed consent was obtained from all patients before enrollment in the study. The trial was registered prior to patient enrollment at Chinese Clinical Trial Registry (Registration NO. ChiCTR1900026194; Principal investigator, Zhiyu Geng; Date of registration: Sep 26, 2019). This manuscript adheres to the applicable CONSORT guidelines.

### Study population

Female patients aged 18–65 years old with American Society of Anesthesiologists (ASA) physical status I-II, scheduled for elective laparoscopic gynecological surgery for a benign indication were assessed for eligibility. Exclusion criteria were ASA physical status III or more, pre-existing hepatic (liver enzymes more than two times normal values), renal (estimated glomerular filtration rate < 60 mL/min/1.73m^2^) or bowel disease, current use of corticosteroid or opioid analgesic, allergy or contraindication to any drug used in the study, pregnant, breasting, or refuse to participate the study.

### Randomization and blinding

This is a randomized patient and assessor-blinded controlled trial. After written informed consent, participants were randomly assigned to Study group or Control group. Randomization was carried out using a computer-generated random number list on a 1:1 ratio by statistician, and group assignment was performed by opaque, sealed envelopes prepared by a research nurse not involved in the study. All outcome assessments and perioperative data collection were performed by a research assistant not involved with patients care and blinded to the group allocation throughout the study period.

### Anesthetic technique

On arrival in the operating room, routine monitoring included non-invasive blood pressure, pulse oximetry, electrocardiogram and bispectral index (BIS) were applied in both groups. All patients received general anesthesia and mechanical ventilation. Induction of anesthesia was performed with midazolam 0.03 mg/kg, propofol 1.5-2 mg/kg and remifentanil target effect site concentration 3 ng/ml. Rocuronium 0.6 mg/kg was administered to facilitate the Supreme laryngeal mask insertion. Anesthesia maintenance was achieved with continuous infusion of propofol 5–6 mg/kg/h and remifentanil 3-4 ng/ml, titrated to maintain mean blood pressure within 20% of baseline, and BIS values between 40 and 60. Oxygen and air were administered in a ratio of 1:1 and ventilation was controlled to maintain an end-tidal carbon dioxide partial pressure between 35 and 55 mmHg. The maintenance fluid was Lactate Ringer’s solution. Intraoperative fluid administration was based on change in hemodynamic parameters throughout the surgery. Dual antiemetic agents were given, including dexamethasone 5 mg after induction and tropisetron 5 mg prior the end of the surgery. All procedures were performed by two senior gynecological surgeons to ensure conformity. On completion of surgery, patients were extubated and transferred to the post-anesthesia care unit (PACU) for observation.

### Interventions

Patients in the Study group received multimodal pain regimen, including oral acetaminophen 650 mg and oral gabapentin 600 mg 2 h before surgery, 1% ropivacaine 10 ml trocar areas infiltration after skin closure, two doses of intravenous flurbiprofen 50 mg (at the end of surgery and 6 h postoperatively), oral acetaminophen (650 mg every 8 h) and celecoxib (200 mg every 12 h) on postoperative day (POD) 1–2. Following elements of ERAS program were also applied: no mechanical and oral bowel preparation, clear carbohydrate beverage (5 ml/kg) allowed up to 2 h before surgery, fluids limited to less than 2 L of crystalloids, normothermia maintained, early oral diet and early ambulation.

Patients in the Control group received our conventional analgesia. A single dose of intravenous flurbiprofen 50 mg was administered 30 min before the end of the procedure. Preoperative intervention included routine bowel preparation and no oral fluid was allowed prior to surgery.

During the first 48 h, all patients were provided morphine patient-controlled analgesia (PCA) for rescue analgesia (no basal infusion, 1 mg bolus with a 6-min lockout, and started in the PACU). Pain intensity was assessed using an 11-point numeric rating scale (NRS: 0 meant no pain, and 10 was the worst pain imaginable) by an investigator blinded to group allocation. Additional IV morphine was given for NRS pain score ≥ 4. Sedation levels were assessed using the Ramsay sedation scale (1 = agitated and uncomfortable, 2 = co-operative and orientated, 3 = can follow simple directions, 4 = asleep but strong response to stimulation, 5 = asleep and slow response to stimulation, 6 = asleep and no response to stimulation). Over sedation was defined as a sedation score ≥ 4 [11]. Nausea or vomiting was treated with tropisetron or metoclopramide. Rescue antiemetics were administered on the following conditions: two or more episodes of vomiting or retching, any nausea lasting for more than 30 min, a ‘severe’ degree of nausea or whenever treatment was requested by the patient.

### Outcome measures

The primary outcome was QoR-40 score on POD 2. The questionnaire contains 40 questions examining five domains of patient recovery: emotional status, physical comfort, psychological support, physical independence, and pain. Each question uses a 5-point Likert scale as follows: none of the time, some of the time, usually, most of the time, and all the time. The global QoR-40 scores range from 40 to 200, representing very poor to outstanding quality of recovery. QoR-40 is the most common used evaluation method of postoperative recovery and has been widely validated for different type of surgery and anesthetic technique [12–14].

The secondary outcomes included NRS scores, cumulative morphine consumption, requirement for rescue analgesic, time to first ambulation, time to tolerate solid diet and time to return of bowel function (passage of flatus). Serum C-reactive protein (CRP) at POD 2, and any adverse events such as PONV, over sedation, dizziness and fever (body temperature ≥ 38 °C) were also documented.

All data were collected by an investigator who was blinded to the group assignment and not involved in patient’s perioperative care. The 48 h observation period started at the time of removal of the laryngeal mask airway. The researcher assessed the patients in the PACU, at 2 h, 6 h, 24 h and 48 h postoperatively.

### Statistical analysis

The Shapiro-Wilk and Kolmogorov-Smirnov tests were used to test the hypothesis of normal distribution. Normally distributed continuous variables were described as means ± standard deviation (SD), and analyzed with a two-sided independent *t-*test. Non-normally distributed variables were described as median (interquartile range [IQR]), and analyzed using the Mann–Whitney *U* test. Categorical variables were described as number (percentage) and analyzed using the Chi square test or Fisher’s exact test as appropriate. For postoperative cumulative morphine consumption and NRS scores, a Bonferroni correction for multiple between-group comparisons was used to control for false positive rates.

All statistical analysis was performed using the SPSS 22.0 software (SPSS, Inc., Chicago, Illinois, USA). A two-sided *P* value less than 0.05 was considered statistically significant. For cumulative morphine consumption and NRS scores, 4 comparisons were adjusted and *P* value less than 0.05/4 = 0.013 was considered statistically significant.

The sample size was calculated according to data from previous studies [14–17]. Among these studies, the standard deviations or interquartile ranges of the postoperative global QoR-40 score were 26 in female patients undergoing diverse surgery, 19 or 21 in gynecological surgical patients,17 or 22 in thyroid surgical patients. A 10-point difference between groups represents a clinically relevant improvement in quality of recovery [14–16]. Assumed a common standard deviation of 20, a sample size of 64 patients per group was estimated to achieve 80%power to detect a difference for the two study groups at α 0.05 significance level. To allow for a possible10% dropout rate, 140 subjects were recruited and randomized.

## Results

**Fig. 1:**
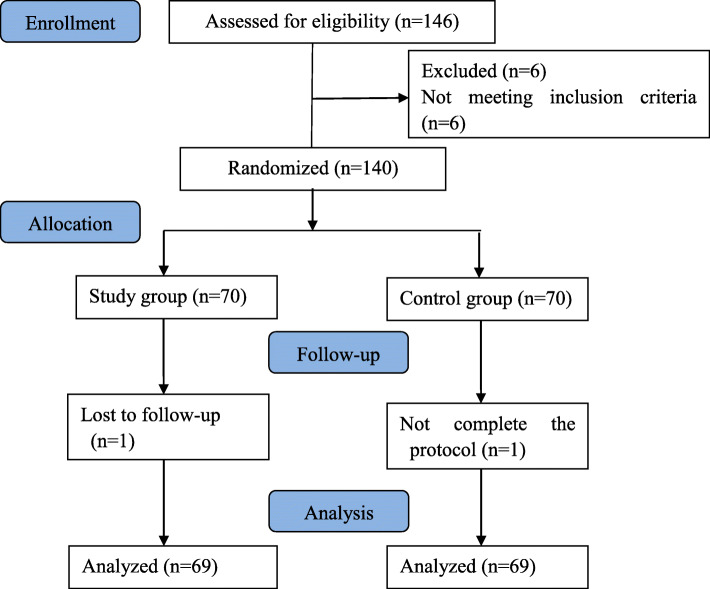
CONSORT flow diagram

Of the one hundred and forty-six patients assessed for eligibility, six patients were excluded because of not meeting inclusion criteria. One hundred and forty subjects were randomized and 138 completed the study. Two patients were excluded after enrollment due to lost to follow-up (*n* = 1 patient, Study group) or protocol violation (*n* = 1 patient, Control group). The CONSORT flow diagram was presented in Fig. 1.

The patient demographics and surgical data were not significantly different between the two groups (Table 1). The primary outcome of the global QoR-40 score at POD 2 was not significantly different between two groups. The mean QoR-40 scores was 184.8 ± 13.0 in the Study group and 182.7 ± 12.4 in the Control group (*P* = 0.338). Nonetheless, when the five dimensions was analyzed separately, the score in the pain dimension was higher in the Study group (32.1 ± 3.0 vs. 31.0 ± 3.2, *P* = 0.033) (Table 2).

**Table 1:** Patient characteristics and surgical data

	Study Group (*n* = 69)	Control Group (*n* = 69)	*P* value
Age (year)	39.7 ± 10.5	40.3 ± 11.3	0.762
Height (cm)	162.2 ± 4.8	160.7 ± 5.7	0.083
Weight (kg)	63.0 ± 11.1	60.6 ± 9.7	0.184
BMI (kg/m^2^)	23.9 ± 4.0	23.5 ± 3.4	0.468
ASA physical status I/II (n)	38/31	41/28	0.606
Apfel score for PONV risk (n/%)			0.816
1	0	0	
2	4 (5.8)	3 (4.3)	
3	53 (76.8)	56 (81.2)	
4	12 (17.4)	10 (14.5)	
Average number of risk scores	3.1 (0.5)	3.1 (0.4)	0.850
Type of surgery (n/%)			0.336
LSO	30 (43.5)	36 (52.2)	
LM	14 (20.3)	8 (11.6)	
LH ± LS	25 (36.2)	25 (36.2)	
Anesthesia time (min)	72 (58.5–89.5)	80 (58.5–106)	0.112
Operation time (min)	55 (40.5–71.5)	60 (42–86.5)	0.083

Data are presented as mean ± standard deviation, median (interquartile range), or number of patients (%) where appropriate

*Abbreviations*: *ASA* American Society of Anesthesiologists, *BMI* Body mass index, *LSO* Laparoscopic salpingo-oophorectomy, *LH* Laparoscopic hysterectomy, *LS* Laparoscopic salpingectomy, *LM* Laparoscopic myomectomy

**Table 2:** Dimensions of the QoR-40 Questionnaire on POD 2

	Study Group (*n* = 69)	Control Group (*n* = 69)	Mean difference (95% CI)	*P* value
Global QoR-40 score	184.8 ± 13.0	182.7 ± 12.4	2 (−1, 7)	0.338
Emotional state	40.3 ± 5.2	39.7 ± 4.5	1 (0, 2)	0.474
Physical comfort	55.3 ± 4.4	54.7 ± 4.1	1 (−1, 2)	0.446
Psychological support	33.5 ± 2.5	33.8 ± 2.2	0 (0, 0)	0.594
Physical independence	23.7 ± 2.3	23.5 ± 2.5	0 (0, 0)	0.575
Pain	32.1 ± 3.0	31.0 ± 3.2	1 (0, 2)	0.033

Data are presented as mean ± standard deviation

*Abbreviations*: *POD* Postoperative day

Comparing with the Control group, the NRS pain scores were significant lower in the Study group at all time points postoperatively (*P* = 0.000). In PACU, more patients in the Control group required rescue analgesics for moderate pain (27.5% vs. 5.8%; *P* = 0.0006). Total morphine consumption throughout the study period was significantly less in the Study group when compared with the Control group [3 (1–4.5) vs. 5 (3–7.5), *P* = 0.033].

There was no difference between the two groups regarding the incidence of PONV [(15.9% vs. 20.3%); *P* = 0.507)]. In addition, the rate of rescue antiemetic medication, adverse events such as fever and dizziness were also similar between study groups (Table 3).

**Table 3:** Postoperative pain management and adverse events

	Study Group (*n* = 69)	Control Group (*n* = 69)	Median difference (95% CI)	*P* value
NRS scores at PACU admission	1 (1–2.5)	4 (3–5)	−3 (−3, − 2)	0.000 *
NRS at 2 h	1 (1–2)	3 (3–3)	−2 (− 2, − 2)	0.000 *
NRS at 6 h	1 (1–2)	2 (2–3)	−1 (− 2, − 1)	0.000 *
NRS at 24 h	1 (1–1)	2 (1–3)	−1 (− 1, − 1)	0.000 *
NRS at 48 h	0 (1–1)	1 (1–1)	−1 (− 1, − 1)	0.000 *
Rescue analgesics in PACU (n/%)	4 (5.8)	19 (27.5)		0.0006
Total morphine consumption in PACU (mg)	1 (1–2)	2 (2–4)	−1 (− 2, − 1)	0.000 *
Total morphine consumption in 24 h (mg)	2 (1–3.5)	4 (3–7)	− 2 (− 3, − 1)	0.000 *
Total morphine consumption in 48 h (mg)	3 (1–4.5)	5 (3–7.5)	−2 (− 3, − 1)	0.000 *
PACU sedation (n/%)	5 (7.2)	0		0.068
PONV 0-48 h (n/%)	11 (15.9)	14 (20.3)		0.507
Rescue antiemetics 0-48 h (n/%)	2 (2.9)	2 (2.9)		0.612
Postoperative fever (n/%)	3 (4.3)	5 (7.2)		0.716
Postoperative dizziness (n/%)	8 (11.6)	11 (15.9)		0.459

Data are presented as median (interquartile range)

*Abbreviations*: *NRS* Numeric rating scale, *PACU* Post-anesthesia care unit, *PONV* Postoperative nausea and vomiting, *CI* Confidence interval

*P < 0.013, the Mann-Whitney U test with Bonferroni correction

Compared to the Control group, median time to ambulation [5.0 (3.3–7.0) h vs. 6.5 (5.0–14.8) h; *P* = 0.003] and time to return of bowel function [14.5 (9.5–19.5) h vs.17 (13–23.5) h; *P* = 0.008] were shorter, CRP values on POD2 was lower [4(3–6) ng/ml vs. 5 (3–10.5) ng/ml; *P* = 0.022], and patient satisfaction was higher (9.8 ± 0.5 vs. 8.8 ± 1.2, *P* = 0.000) in the Study group. (Table 4).

**Table 4:** Postoperative recovery data

	Study Group (*n* = 69)	Control Group (*n* = 69)	Median difference (95% CI)	*P* value
Time to ambulation (h)	5.0 (3.3–7.0)	6.5 (5.0–14.8)	−2.0 (−3.0, −0.5)	0.003
Time to first solid diet (h)	7.0 (6–8.0)	19.0 (16.0–21.0)	−12.0 (− 13.0, − 10.5)	0.000
Time to removal of urinary catheter (h)	8.0 (5.5–48)	26.5 (23.0–47.0)	−15.0 (− 19.0, − 1.0)	0.014
Time to return of bowel function (h)	14.5 (9.5–19.5)	17.0 (13.0–23.5)	−3.0 (− 5.5, − 1.0)	0.008
CRP on POD2 (ng/ml)	4.0 (3.0–6.0)	5.0 (3.0–10.5)	−1.0 (− 2.0, 0)	0.022

Data are presented as median (interquartile range) or number of patients (%)

*Abbreviations*: *CRP* C-reactive protein, *POD* Postoperative day

## Discussion

This prospective randomized clinical trial investigated the impact of multimodal analgesia, as a part of enhanced recovery protocol, on quality of recovery after laparoscopic gynecological surgery. Our results revealed similar global QoR-40 score on POD 2 in multimodal analgesic group, although better pain relief, lower morphine consumption, earlier ambulation, and faster bowel function recovery were achieved when compared to conventional opioid analgesic group.

The QoR-40 questionnaire is a valid and reliable measurement used to assess the degree of recovery after different surgical types and anesthetic techniques. It provides a patient-assessed health status through five dimensions. A negative association between the global score and duration of hospital day was demonstrated in different types of surgery [14]. Contrary to our expectation, we found no significant difference between two groups regarding QoR-40 score, although lower pain score and decreased opioid use were achieved in the study group. Similar to our findings, other clinical trials also had negative outcomes. Kamiya and colleagues [18] evaluated the effect of pectoral nerve block on quality of recovery after breast cancer surgery. Although the pectoral nerve block improved pain score at 6 h postoperatively, the QoR-40 score on POD 1 was not improved. Fujimoto and colleagues [19] demonstrated no improvement in quality of recovery or postoperative analgesia for patients received single-shot posterior quadratus lumborum blockade (QLB) after laparoscopic gynecological surgery. The median QoR-40 score was 154 (133–168) in the QLB group and 158 (144–172) in the control group (*P* = 0.361) respectively. Another study also showed no significant difference in QoR-40 scores when utilizing the TAP block on laparoscopic hysterectomy patients. The overall QoR-40 score was 168 (125–195) in the transverses abdominis plane (TAP) block versus 169.5 (116–194) in the no-block group [20].

Postoperative pain can potentially influence QoR-40 scores [17, 21]. Two studies have suggested that preoperative TAP block was associated with reduced pain intensity and better quality of recovery in patient undergoing laparoscopic gynecological surgery, an inverse relationship between 24 h opioids consumption and time to discharge readiness was also demonstrated [22, 23].

Multimodal analgesic strategy with nonopioids analgesic results in better analgesia, less opioid use, and enhanced recovery. Beneficial effects after laparoscopic surgery have been shown in previous studies. Kell and colleagues [24] performed a cohort study on laparoscopic colorectal resection patients, finding significantly less intraoperative fentanyl, lower PACU pain scores and shorter length of stay in multimodal pain management including TAP block and local peritoneal infiltration with long-acting liposomal bupivacaine. Ng and colleagues [25] conducted a retrospective study involving one hundred and fifty-eight patients who underwent laparoscopic sleeve gastrectomy. They found that multimodal analgesic protocol reduced incidence of opioid related adverse events, provided effective pain relief even with less postoperative opioid use.

In our study, we used a multimodal analgesic protocol including acetaminophen, gabapentin, ropivacaine, NSAIDs and dexamethasone in study group. Wound infiltration with local anesthetics decreased abdominal pain, and systemic inflammatory pain may be controlled by NSAIDs and corticosteroids. Consequently, we found lower pain score, less opioid use, lower CRP value, shorter time to ambulation, and faster bowel function recovery in multimodal analgesic group.

However, we failed to demonstrate any improvement in QoR-40 score when utilizing multimodal analgesic protocol on laparoscopic gynecological patients. We speculated that the negative result might be attributed to overall low pain scores in minimally invasive laparoscopic surgeries. Our results showed that pain intensity was maximal in the first 6 h after surgery, and gradually declined to low levels within the first 2 days of surgery. Although one point difference in pain score was statistically significant, it was likely not clinically significant thus make difference in quality of recovery.

Additionally, it is important to note that, not only nociceptive stimulus from tissue trauma, but also other factors such as socio-culture and individual characteristics may affect subjective pain perception. Wolmeister and colleagues [26] demonstrated that individuals with elevated preoperative emotional stress present higher postoperative pain levels. Person and colleagues [27] suggested that women with high stress-coping abilities have a better outcome in general well-being than women with low stress-coping capacity. Among five dimensions of the QoR-40, pain, physical comfort, and physical independence were mainly affected by surgery, while emotional state and psychological support were likely to be influenced by individual characteristics.

There are some limitations to this study. First, this was a single-center study, and our cohort was relatively young. We excluded patients with significant comorbidities or chronic pain conditions. Hence, potentially greater benefits may have been found if more diverse patients had been included. Secondly, since preoperative QoR-40 scores may not be suitable for comparing recovery after surgery, multiple postoperative evaluations would be better for obtaining meaningful results. Future studies should focus perioperative psychological factors to further improve postoperative quality of recovery.

## Conclusions

For minimally invasive laparoscopic gynecological surgery, multimodal analgesia based enhanced recovery protocol offered better pain relief, lower opioid use, earlier ambulation, faster bowel function recovery and higher patient satisfaction, although no improvement in QoR-40 score was found.

## Data Availability

The datasets generated and/or analyzed during the current study are not publicly available due to patient confidentiality but are available from the corresponding author on reasonable request.

## Abbreviations

ERAS: Enhanced recovery after surgery
PONV: Postoperative nausea and vomiting
PCA: Patient-controlled analgesia
PACU: Post-anesthesia care unit
VAS: Visual analogue scale
QoR: Quality of recovery
